# Comparative Evaluation of DNA Extraction Workflows for Efficient Recovery of pBI143 from Wastewater

**DOI:** 10.1007/s12560-026-09689-z

**Published:** 2026-04-17

**Authors:** Mustafa Ali, Ocean Thakali, Oladele Idris, Samendra Sherchan

**Affiliations:** 1 Center for research excellence in wastewater-based epidemiology, Morgan State University, Baltimore, USA; 2 Bioenvironmental Science program, Morgan State University, Baltimore, USA

**Keywords:** CrAssphage, Magnetic nanoparticles, pBI143, PEG precipitation, PMMoV, ToBRFV

## Abstract

This study aimed to compare the performance of polyethylene glycol (PEG) precipitation, and Nanotrap^®^ Microbiome magnetic particle capture workflows for recovering novel fecal marker, pBI143 from 12 wastewater samples collected across six treatment plants in Maryland, USA. Quantitative PCR (qPCR) was used to quantify marker abundance. The Nanotrap workflow yielded significantly higher concentration of pBI143 compared to PEG precipitation workflow (*p* < 0.05). The Nanotrap workflow used in the study utilized both magnetic nanoparticles A and B, rather than magnetic nanoparticle A alone, highlighting the necessity of optimization based on the intended targets for enhanced recovery. The extracted total nucleic acids by the Nanotrap workflow, were further analyzed to quantify other fecal markers, crAssphage, tomato brown rugose fruit virus (ToBRFV), and pepper mild mottle virus (PMMoV). No significant differences in the concentrations of pBI143, crAssphage, and ToBRFV (*p* > 0.05) were observed, whereas the concentration of PMMoV was significantly lower than that of the three fecal markers (*p* < 0.05). Based on the concentration alone, pBI143, ToBRFV, and crAssphage were found to be a better alternative to PMMoV as an endogenous fecal marker.

## Introduction

Wastewater-based epidemiology (WBE) has emerged as a critical tool for public health monitoring, especially in the wake of the COVID-19 pandemic (Verani et al., 2024). However, fluctuations in wastewater flow and storm events can impact the accurate estimation of analyte concentrations. To account for these variations, normalization using endogenous fecal markers is a commonly employed strategy for the reliable interpretation of wastewater surveillance data (Greenwald., 2021). Therefore, central to WBE is the ability to extract, quantify, and analyze both genetic material of target of interest and endogenous fecal marker from complex wastewater matrices. Since recovery of bacteria, virus and protozoan pathogens from wastewater have been shown to be heavily influenced by the selected concentration and extraction workflows, careful selection of workflow is critical to ensuring accurate detection and quantification (Lemarchand et al., 2005; Ahmed et al., 2020, Morales et al., 2003).

Recently, a cryptic plasmid pBI143 has been revealed to be the most abundant genetic element in the human gut microbiome of individuals from industrialized countries with minimal detection in fecal samples from non-human animals (buffalo, cat, chicken, cow, deer, dog, elk, fish, goat, human, insect, macaques, mouse, panda, pig, rat, sheep, termite, vole, whale, boar, yak, and zebu) (Fogarty et al., 2024). pBI143 has also been demonstrated to be stable in wastewater with higher concentration than other viral fecal markers, including crAssphage, a double-stranded DNA (dsDNA) bacteriophage, and pepper mild mottle virus (PMMoV), an RNA plant virus, as well as human-specific Bacteroidales markers (HF183, Lachno3, and gyrB (Malla et al., 2024, 2025; Liu et al., 2025; Johnson et al., 2024; Fogarty et al., 2024). Overall, this suggests pBI143 holds great potential as an endogenous fecal marker for normalization purpose in WBE and as a human-specific fecal marker in microbial source tracking. However, only three studies have evaluated various workflows on optimum recovery of pBI143 from wastewater. Malla et al., 2024 reported higher recovery of pBI143 using polyethylene glycol (PEG) precipitation compared to filtration and centrifugation techniques. Similarly, concentrating wastewater using PEG also resulted in higher recovery of pBI143 compared to filtration technique in a study conducted in Australia (Liu et al., 2025). In contrast, only one study has utilized magnetic nanoparticles for concentrating pBI143 in wastewater, reporting greater recovery rates than those achieved with skimmed milk flocculation, ultrafiltration, PEG precipitation, and filtration methods (Johnson et al., 2024). The use of magnetic nanoparticles for concentrating analytes in wastewater is a relatively newer technique compared to others and is based on the principle that magnetic beads will bind to bacterial and viral components such as capsid proteins and glycoproteins via electrostatic and hydrophobic interactions (Andersen et al., 2023). However, the PEG precipitation workflow used in the study involved removal of pellets prior to PEG precipitation despite acknowledging pBI143 to be present mostly in solid fraction of wastewater (Johnson et al., 2024). A fair comparison between the two optimal techniques for concentrating pBI143 is still lacking, and we hypothesize that the magnetic nanoparticle protocol can be further optimized to achieve greater recovery. For example, concentrating wastewater using magnetic nanoparticles and extracting nucleic acids with an automated instrument such as the KingFisher Apex purification system (ThermoFisher Scientific, Waltham, MA, USA) can reduce human error and variability associated with manual sample processing.

This study aimed to compare the recovery efficiency of pBI143 using PEG precipitation and automated magnetic nanoparticle workflows. We also compared the concentration of pBI143 to that of crAssphage, pepper mild mottle virus (PMMoV), and tomato brown rugose fruit virus (ToBRFV), an RNA virus in wastewater. crAssphage is one of the most abundant viruses in the human gut microbiome and is widely used as a human-fecal marker. PMMoV is a well-established fecal marker (Kitajima et al., 2018) and ToBRFV has been recently proposed as a human-associated fecal marker due to its high abundance in human feces and wastewater (Natarajan et al., 2023). Ultimately, this work seeks to provide insights into the best workflow to recover pBI143 marker from wastewater and assess its suitability as a fecal marker.

## Materials and Methods

### Wastewater Sample Collection

Twelve wastewater samples were collected between March and April 2025 from six different wastewater treatment plants (WWTPs) located in Maryland and Washington D.C., USA. Samples were collected in sterile containers and transported on ice to the laboratory.

### Concentration and Nucleic Acids Extraction

To evaluate extraction efficiency, three DNA extraction workflows were performed in parallel: (1) Direct extraction, (2) PEG precipitation, and (3) Automated Nanotrap capture.

#### Direct Extraction Workflow

A 250 µL aliquot of untreated wastewater was subjected to direct DNA extraction using the Qiagen DNeasy PowerSoil kit (Qiagen, Hilden, Germany), following the manufacturer’s instructions. DNA was eluted in 100 µL and stored at −80 °C until further analysis. Here, the concentration of pBI143 quantified using this workflow was considered as the “true” concentration of pBI143 in wastewater (Johnson et al., 2024).

#### PEG Precipitation Workflow

A 40 mL aliquot of wastewater was mixed with 4.0 g of polyethylene glycol 8000 (PEG 8000; ThermoFisher Scientific, Waltham, MA, USA; Cat. No. 81268) and 0.94 g sodium chloride (ThermoFisher Scientific, Cat. No. S271-500). The mixture was vortexed for 10 min at room temperature and centrifuged at 4,250 × g for 99 min at 4 °C. The supernatant was reduced by decanting the excess, leaving behind a final volume of 5 mL. This concentrate was subjected to a second centrifugation at 4,250 × g for 5 min at 4 °C. The supernatant was then completely discarded, and the resulting pellet was resuspended in 500 µL of nuclease-free water. A 250 µL aliquot of PEG concentrate was then used to extract 100 µL of DNA using the Qiagen DNeasy PowerSoil kit (Qiagen), following the manufacturer’s instructions. The PEG precipitation procedure was adapted from previously described protocols (Malla et al., 2025).

#### Nanotrap Microbiome Capture Workflow

A 4,800 µL aliquot of wastewater was concentrated and total nucleic acids was extracted using the Nanotrap Microbiome Combined; 10 mL Automated Protocol (Ceres Nanosciences., 2022) on an automated Kingfisher Apex system. This protocol utilizes the MagMAX Microbiome Ultra nucleic acid isolation kit (ThermoFisher Scientific) to yield 100 µL of total nucleic acids. The Nanotrap Microbiome Combined protocol utilizes both Nanotrap Microbiome particles A and B (Ceres Nanosciences Inc., Manassas, VA, USA) for enhanced capture and concentration of different classes of microorganisms from wastewater (Ceres Nanosciences, 2022).

### Complementary DNA (cDNA) Synthesis

Complementary DNA (cDNA) was synthesized using the High-Capacity cDNA Reverse Transcription Kit (Applied Biosystems, USA) as per the manufacturer’s instructions. Each 20 µL reaction included 10 µL of extracted RNA, 2 µL of 10× RT Buffer, 0.8 µL of 25× dNTP Mix (100 mM), 2 µL of 10× RT Random Primers, 1 µL of MultiScribe™ Reverse Transcriptase (50 U/µL), 1 µL of RNase inhibitor and 3.2 µL of nuclease-free water.

### Quantitative Polymerase Chain Reaction (qPCR)

All qPCR amplifications were carried out using a Bio-Rad CFX96 thermal cycler (Bio-Rad, California, USA) in a final reaction volume of 20 µL. Each qPCR reaction mixture contained 10 µL of PerfeCta qPCR ToughMix, Low ROX (Quantabio, MA, USA), 900 nM of each forward and reverse primer, 250 nM of probe, 2.5 µL of template DNA, and PCR-grade water. Primers and probes previously designed by Stachler et al., (2017) for crAssphage, Fogarty et al., (2024) for pBI143, Zhang et al., (2006) for PMMoV and international Seed Health Initiative for Vegetable Crops (2020) for ToBRFV (ISHI-Veg, 2020).

The thermal conditions were 95 °C for 3 min, followed by 45 amplification cycles of 95 °C for 15 s and 60 °C for 30 s. All qPCR reactions were performed in duplicate. No-template control comprised of PCR grade water. For positive control and standard curve generation, six ten-fold serial dilutions of synthetic gBlock gene fragments (Integrated DNA Technologies, Coralville, USA) prepared using Easy Dilution solution (Takara Bio, Kusatsu, Japan; Cat. No. 9160) were used. To assess potential PCR inhibition, amplification efficiencies and standard curve performance were evaluated for all assays. The standard curve equations were as follows: crAssphage: *y* = − 3.4176*x* + 40.751, pBI143: *y* = − 3.4608*x* + 40.492, PMMoV: *y* = − 3.2524*x* + 42.998, and ToBRFV: *y* = − 3.4038*x* + 38.98. All standard curves demonstrated R² values greater than 0.99 with amplification efficiencies within the acceptable range (90–110%), indicating reliable amplification performance. Additionally, crAssphage concentrations obtained across workflows were within previously reported ranges for raw wastewater (Sabar et al., 2022), suggesting minimal PCR inhibition in the analyzed samples.

### Statistical Analysis

The concentrations of all fecal markers log_10_ transformed prior to statistical analysis. Paired t-test and Two-Way Repeated Measures ANOVA were performed using RStudio R version 4.4.0 (Core Team, 2024). A p value of < 0.05 was considered significant for all analyses. Recovery efficiency (%) was calculated by dividing the concentration obtained from the PEG precipitation method by the concentration obtained from the direct extraction method and multiplying it by 100.

## Results and Discussion

### Comparison of Workflows for Recovery of pBI143

First, crAssphage was quantified in all of the wastewater samples as an endogenous fecal marker to assess recovery of microbial targets and/or presence of significant PCR inhibitors. crAssphage was detected in 100% (36/36) of the samples tested regardless of the workflow used. The average concentration of crAssphage recovered using the direct extraction, PEG precipitation, and Nanotrap workflows were 9.30 ± 0.40, 8.59 ± 0.33, and the 9.71 ± 0.40 log_10_ copies/L, respectively. A recent review on crAssphage in water environment (Sabar et al., 2022) has reported crAssphage concentration in raw wastewater of high-income countries such as USA, UK, Australia, New Zealand, and Spain ranged from 7.4 to 10.0 log_10_ copies/L, which suggests negligible presence of PCR inhibitors and efficient recovery of DNA targets.

Similarly, pBI143 was also detected in 100% (36/36) of the wastewater samples tested using all three workflows. The average concentration of pBI143 using the direct extraction, PEG precipitation, and Nanotrap workflows were 9.02 ± 0.60, 8.55 ± 0.75 and 9.68 ± 0.70 log_10_ copies/L, respectively. Assuming that the concentration of pBI143 yielded using direct workflow to be the actual concentration of pBI143 in our wastewater samples (Johnson et al., 2024), the recovery efficiency of PEG workflow was found to be 43.8 ± 42.44%. In complex matrices such as wastewater, a recovery of 1% is considered acceptable (Haramoto et al., 2018). Therefore, our findings align with the results of past studies (Liu et al., 2025; Malla et al., 2025) that reported PEG precipitation as the most effective concentration technique for recovering pBI143 from wastewater. It should be noted that our PEG precipitation workflow used a lower centrifugal force compared to 10,000–12,000 ×g used by the past studies (Liu et al., 2025; Malla et al., 2025). Despite the lower centrifugal force applied, our results demonstrate that a standard benchtop centrifuge, commonly available in smaller laboratories, is sufficient for effective recovery of pBI143 from wastewater. This finding can be particularly important for resource limited settings where high speed ultracentrifuges may not be readily available.

**Fig. 1:**
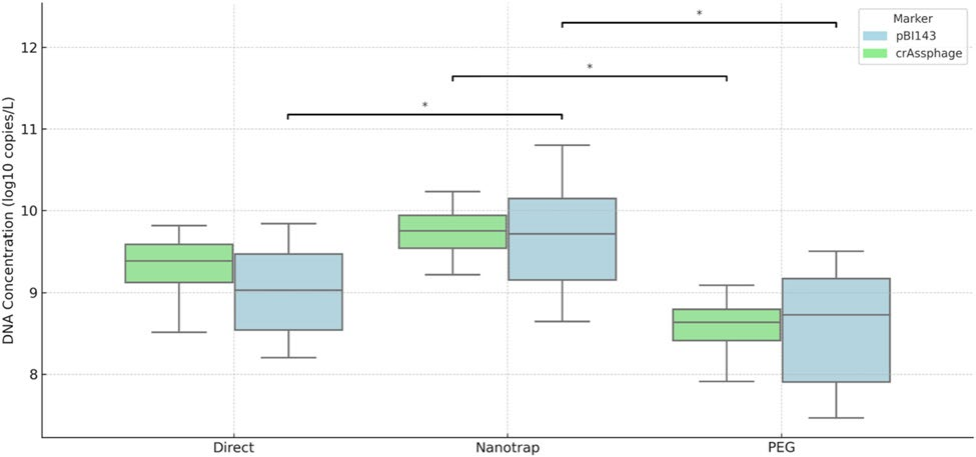
Concentration of crAssphage and pBI143 recovered using three different workflows. The asterisk (*) sign signifies significance at *p* < 0.05

As shown in Fig. 1, the Nanotrap workflow yielded significantly higher concentration of crAssphage and pBI143 compared to both direct and PEG precipitation workflows (paired t-test, *p* < 0.05). The lower concentrations observed using the direct extraction workflow compared to the Nanotrap method may be explained by differences in processed sample volume and the absence of a concentration step. The direct workflow processed only 250 µL of untreated wastewater without prior enrichment, whereas the Nanotrap workflow processed 4.8 mL and incorporated an active magnetic capture step before nucleic acid extraction. Analyzing a small, untreated aliquot may underrepresent particle-associated targets. In contrast, the Nanotrap microbiome protocol utilizes magnetic particles designed to bind microbial cells and associated nucleic acids, thereby enriching both free and particle-associated genetic material prior to extraction. Additionally, differences in extraction chemistry between the two commercial kits may have contributed to variation in DNA yield and purity, further influencing quantification results.

Although the Nanotrap workflow processes a smaller input volume (4.8 mL) compared to PEG precipitation (40 mL), which theoretically may result in a higher sample-based limit of detection, the significantly higher concentrations observed in this study suggest that improved capture efficiency compensates for the reduced processed volume. Assuming similar qPCR detection limits across workflows, the approximately eight-fold difference in processed volume would theoretically translate to a proportional difference in method detection sensitivity. Therefore, in extremely low-prevalence settings where target concentrations approach the assay detection limit, PEG precipitation may provide a theoretical advantage due to the larger processed volume. However, a formal experimental determination of method-specific limits of detection was not performed in this study and warrants further investigation.

During PEG precipitation, loss of target DNA can occur during the decantation of the supernatant or resuspension of pellets, especially if the pellet is invisible, or adheres to the tube wall, making it difficult to resuspend. Additionally, PEG is known to co-precipitate PCR inhibitors such as humic substances, polysaccharides, and other organic compounds commonly present in wastewater, which can lead to reduced amplification (Hamza & Leifels, 2023). Additionally, differences in the extraction efficiency of the two kits can lead to variability in both DNA yield and purity, which may impact quantification and contribute to differences in recovery efficiency among workflows.

Our results contrast with the only study that compared direct workflow with magnetic nanoparticle extraction using magnetic nanoparticle A (Ceres Nanosciences) and PEG precipitation and emphasized superior recovery of pBI143 using direct extraction (Johnson et al., 2024). According to the manufacturer of the magnetic nanoparticles, nanoparticle A is recommended for use when targeting viruses such as influenza A, influenza B, respiratory syncytial virus, coronavirus 229E, coronavirus OC43, SARS-CoV-2, Zika virus, Chikungunya virus, dengue virus, pepper mild mottle virus, hepatitis A virus and mpox virus. The same vendor also manufactures nanoparticle B and recommends it for targeting bacteria such as *Escherichia coli*,* Salmonella enterica*,* Campylobacter jejuni*,* Listeria monocytogenes*,* Clostridioides difficile* and crAssphage (Streck, 2025). It is likely that using both magnetic nanoparticles A and B, rather than magnetic nanoparticle A alone, played a major role in the enhanced recovery, highlighting the necessity of optimization based on the intended targets.

The primary aim of wastewater surveillance is to serve as an early warning tool for monitoring infectious disease trends in communities. To be effective in this role, the workflow employed must be rapid, scalable, and capable of generating timely data. The detection sensitivity is a critical parameter when considering application of a workflow as an early warning tool. Early detection of emerging pathogens requires reliable amplification at low target concentrations during the initial phase of outbreaks when pathogen loads in wastewater may be minimal. Although all fecal markers in this study were detected in 100% of samples, the samples evaluated contained relatively high endogenous marker concentrations. Therefore, future studies should assess the limit of detection, reproducibility at low concentrations, and performance in low prevalence scenarios to comprehensively evaluate the suitability of the Nanotrap workflow for early warning applications. In our study, the Nanotrap protocol required approximately 45–60 min for both concentration and nucleic acid extraction, while the PEG precipitation workflow used here, took 3–4 h in total. Some PEG protocols also include an overnight incubation step that can further delay results (Torii et al., 2022). The faster processing time of the Nanotrap workflow represents a critical advantage in urgent public health scenarios, where early detection and response are essential.

### Comparison of Concentration Between pBI143 and Other Fecal Markers

**Fig. 2:**
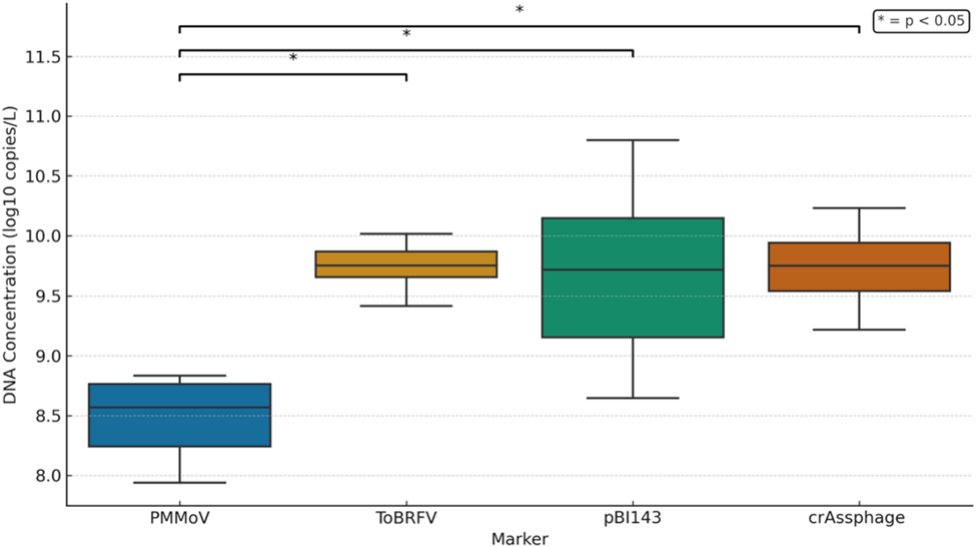
Concentration of various fecal markers recovered using nanotrap extraction workflow. The asterisk (*) sign signifies significance at *p* < 0.05

Since Nanotrap extraction yielded the highest nucleic acid recovery, the extracted total nucleic acids by the Nanotrap workflow, were subsequently analyzed for concentrations of other fecal markers, including ToBRFV and PMMoV. Both ToBRFV and PMMoV were detected in 100% of the wastewater samples using Nanotrap extraction method (*n* = 12). Notably, this is the first study to compare the concentration of pBI143 with ToBRFV. The average concentration of ToBRFV and PMMoV were 9.73 ± 0.24 and 8.44 ± 0.42 log₁₀ copies/L, respectively. No significant differences in the concentrations of pBI143, crAssphage, and ToBRFV (*p* > 0.05) were observed, whereas the concentration of PMMoV was significantly lower than that of the three fecal markers (*p* < 0.05) (Fig. 2).

An ideal fecal marker should consistently exhibit high concentrations in wastewater to allow for robust and reliable detection even at low levels of fecal contamination (Thakali et al., 2025). High concentration of a fecal marker in wastewater also points towards minimal degradation and supports improvement in sensitivity across a wide range of wastewater conditions, and enhance quantification accuracy by reducing the likelihood of non-detection due to dilution. Our finding of higher concentration of ToBRFV compared to that of PMMoV is in consistent with the observations made by Johnson et al., (2025) who also reported significantly higher abundance of ToBRFV compared to the PMMoV in wastewater samples in California, USA. We suggest that this may be due to the broader host range of ToBRFV, which infects both tomatoes and peppers (Salem et al., 2023), whereas PMMoV primarily infects peppers only. As a result, ToBRFV may be more prevalent in commonly consumed foods, leading to high exposure and subsequent excretion in human feces, resulting in greater concentrations than PMMoV.

PMMoV is the most widely used fecal marker in WBE studies due to its high abundance, stability, and detection in a broad range of wastewater samples (Symonds et al., 2018). It is particularly favored in WBE applications targeting RNA viruses, such as SARS-CoV-2, because PMMoV itself is an RNA virus, making it compatible with RNA-based workflows. However, our study findings suggest that PMMoV concentrations are lower than those of pBI143, crAssphage, and ToBRFV when using the Nanotrap workflow, indicating that these three markers are either shed in higher concentrations by humans or possess greater persistence in the wastewater environment. Based on the concentration alone, ToBRFV appears to be a better alternative to PMMoV as an endogenous fecal marker, especially for normalization in RNA-targeting workflows. For DNA-based targets, both pBI143 and crAssphage may serve suitable normalization markers due to their high concentrations. Normalization is an important step in WBE to adjust for dilution effects and better correlate viral concentrations in wastewater with the number of reported cases. Malla et al., (2025) evaluated normalization using all three markers (PMMoV, crAssphage, and pBI143) and reported no consistent improvement in correlation with clinical case data across all settings. Therefore, additional studies conducted at a more granular level—such as on a daily rather than weekly basis—are needed to determine which marker provides the most reliable normalization for robust association with epidemiological trends. Moreover, while pBI143, crAssphage, and ToBRFV show potential as alternative human fecal markers, their specificity must be thoroughly validated, particularly to confirm the absence or consistently low concentrations in non-human fecal sources. This is important to ensure accurate attribution of human fecal contamination. Future studies should focus on validation of host specificity including assessment of potential detection in non-human fecal sources, as well as evaluation of environmental persistence, and geographic consistency to fully assess the suitability of these markers in global WBE and microbial-source tracking efforts.

## Conclusion

This study demonstrated the superiority of Nanotrap microbiome capture workflow compared to PEG precipitation workflow for recovery of pBI143 from wastewater in a relatively shorter processing time. Although the Nanotrap protocol processes a smaller sample volume (4.8 mL) than PEG precipitation (40 mL), the observed higher concentrations suggest enhanced recovery performance. However, the smaller processed volume may influence detection sensitivity in low-abundance scenarios and should be considered when selecting workflows for specific surveillance objectives. Overall, the Nanotrap workflow represents a promising and scalable approach for rapid WBE studies. Since the Nanotrap workflow we used is capable of co-capturing a broad spectrum of DNA and RNA targets, we strongly believe this workflow will be advantageous for use in next generation of high-throughput platforms for WBE studies. Based on the concentration alone, pBI143, ToBRFV, and crAssphage were found to be a better alternative to PMMoV as an endogenous fecal marker. Nevertheless, additional studies are needed to further validate host specificity across diverse animal fecal sources and to assess their persistence under varying environmental conditions. We acknowledge that the wastewater samples analyzed in this study contained relatively high concentrations of fecal markers. As a result, method performance under low-abundance conditions was not evaluated. Since early warning applications depend on reliable detection at low target concentrations, future studies should assess method-specific limits of detection and performance in low-prevalence scenarios to better determine workflow suitability.

## Data Availability

No datasets were generated or analysed during the current study.
